# IMPACT OF DENTAL TRAUMA AND ESTHETIC IMPAIRMENT ON THE QUALITY OF LIFE OF PRESCHOOL CHILDREN

**DOI:** 10.1590/1984-0462/;2017;35;4;00011

**Published:** 2017-09-21

**Authors:** Bruna Miroski Gonçalves, Loraine Fernandes Dias, Carla da Silva Pereira, Marcos Ximenes Ponte, Andréa Cristina Konrath, Michele da Silva Bolan, Mariane Cardoso

**Affiliations:** a Universidade Federal de Santa Catarina, Florianópolis, SC, Brasil.

**Keywords:** Dental aesthetics, quality of life, tooth injuries, Estética dental, Qualidade de vida, Traumatismo dental

## Abstract

**Objective:**

To evaluate the impact of dental trauma and impaired esthetics on the quality of life (QoL) of preschool children and their relatives.

**Methods:**

Study conducted with 192 children aged 2 to 5 years in 11 preschools in Florianópolis, Santa Catarina, South of Brazil. Parents/caregivers completed a questionnaire on quality of life (Brazilian version of the Early Childhood Oral Health Impact Scales - B-ECOHIS), a socioeconomic survey, and then answered specific questions related to dental trauma. The subjects were examined by three accordant examiners (Kappa>0.7). Dental trauma was evaluated on the basis of indexes adopted by the World Health Organization, and esthetic impairment was then classified. Data were descriptively analyzed and put to bivariate analysis by chi-square and Fisher tests, with significance level at 5%.

**Results:**

The prevalence of dental trauma was 62.5% with 15.6% of esthetic impairment. Almost 12% of parents reported impact on the quality of life of their children. Dental trauma was not significantly associated with gender, age or QoL. Crown color change by trauma was associated with esthetic impairment. Also esthetic impairment had a negative impact on QoL (*p*<0.05) and was associated with oral limitations (*p*<0.05).

**Conclusions:**

Esthetic impairment had a negative impact on children’s quality of life, while dental trauma was not associated to it.

## RESUMO

**Objetivo:**

Avaliar o impacto do trauma dental (TD) e do comprometimento estético na qualidade de vida (QV) de pré-escolares e familiares.

**Métodos:**

Este estudo foi realizado com crianças de 2 a 5 anos em 11 pré-escolas de Florianópolis (SC), Brasil. Os pais/responsáveis preencheram a versão brasileira do questionário sobre QV *Early Childhood Oral Health Impact Scales* (B-ECOHIS), um questionário socioeconômico e perguntas específicas sobre TD. As crianças foram examinadas por três examinadores calibrados (kappa>0,7). O TD foi avaliado de acordo com os índices adotados pela Organização Mundial de Saúde (OMS), e o comprometimento estético também foi classificado. Os dados foram analisados descritivamente e submetidos à análise bivariada por qui-quadrado e teste de Fisher, sendo significante *p*<0,05.

**Resultados:**

Dos pré-escolares examinados, 62,5% foram diagnosticados com trauma e 15,6% com comprometimento estético, e somente 12% dos pais relatou que seus filhos apresentam impacto na QV. O TD não se associou a sexo, idade ou QV. A alteração de cor da coroa por TD relacionou-se ao comprometimento estético, e apenas este causou impacto negativo na QV (*p*<0,05) e se associou ao domínio *limitações orais* (*p*<0,05).

**Conclusões:**

O comprometimento estético causou impacto negativo na QV das crianças, e o trauma dental não se associou com a QV das crianças nem de suas famílias.

## INTRODUCTION

Dental trauma (DT) is very common among preschool children.^1^ It is caused by an external impact on a tooth and surrounding tissues. The degree of severity varies according to the extent of the injury.^2^ The prevalence of DT ranges from 9.4 to 41.6% in studies published in Brazil.^1^ ^,^ ^2^ ^,^ ^3^ ^,^ ^4^ ^,^ ^5^ A study from 2016^6^ on the prevalence of trauma in children aged 1 to 4 years, carried out from 2002 to 2012, showed increase in prevalence in the period. In 2002, the prevalence of trauma at ages 1, 2, 3, and 4 was 4.5%, 11.4%, 14%, and 13.9%, respectively; in 2012, the prevalence for the same age groups was 10.4%, 15.9%, 25.7%, and 28.1%. Another similar study^7^ assessed preschool children between 2008 and 2013 and showed a floating prevalence: 31.7% in 2008, 13.3% in 2010, and 22.5% in 2013, which places this issue as an important consideration to be taken. The stage of development of reflexes in preschool age and the lack of motor coordination can lead children to falls, the main cause of DT in this population.^1^

Dental damage by trauma in preschool children may bring physical and psychosocial consequences. It may also result in pain, function loss, emotional stress, adverse effects in developing occlusion, and changes in esthetics.^8^ The most affected teeth are the upper central incisors.^4^ Loss of structure, in cases of coronary fracture, crown color change, and avulsion are possible aesthetic impairments because these are the more evident elements.^9^ The literature describes that anterior teeth with post-traumatic sequelae may hold relation with social and psychological embarrassment such as: being ashamed to smile, difficulty in maintaining emotional balance, problems to eat certain kinds of foods, and impaired hygiene.^10^ Although the International Association of Dental Traumatology (AITD) recommends focusing on the treatment of acute dental injuries, one should not lose sight of other trauma consequences such as crown discoloration.^11^ Parents often question professionals of the field about sequelae mainly for esthetic reasons.^12^ One or more of the changes listed may negatively impact children’s quality of life.^2^ ^,^ ^4^ ^,^ ^13^

The concept of Oral Health-Related Quality of Life (OHRQoL) corresponds to the impact of oral health or diseases on one’s daily functioning, well-being and quality of life.^14^ Oral diseases and diseases in childhood may negatively affect the lives of preschool children, including growth, weight, socialization, self-esteem, learning abilities, and quality of life of their parents.^14^ The Early Childhood Oral Health Impact Scale (ECOHIS) was developed to evaluate the impact of oral health conditions on the quality of life of preschool children (aged 2 to 5 years) and their relatives, and then validated for the Brazilian population.^15^ ^,^ ^16^ ^,^ ^17^

For clinicians, knowing details that help in decision-making for the treatment, such as clinical features associated with psychological and social aspects, is important. Determining the impact caused by dental trauma and associated esthetic impairment on the child’s and relatives’ lives can help in the provision of adequate treatment measures. Thus, information from OHRQoL is essential for both health policy makers - so that one can conduct a suitable assessment of oral health needs - and clinical dentists.^18^

The purpose of this research was, therefore, to verify the occurrence of DT and associated esthetic impairment, and to assess the impact of such changes on the quality of life of preschool children and their relatives.

## METHOD

This study was carried out with children aging 2 to 5 years and enrolled in 11 municipal day care centers in Florianópolis (SC), Brazil. The centers were selected from the total number of pre-schools authorized to be part of the research by the Education Department and that accepted the invitation. A survey of how many children from 2 to 5 years old were regularly enrolled in each preschool was made, so that the number of study subjects in each institution could be proportionally determined. Therefore, the minimum sample size was calculated by an 90% test power and 7% standard error. As there were no prevalence data for the variables comprised in the study, the value of 50% was taken, totaling at least 187 children.

**Figure 1:**
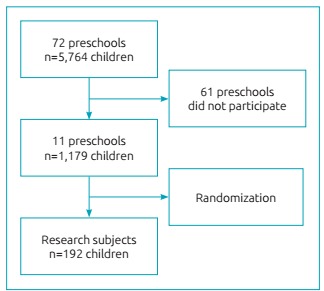
Flowchart for definition of research subjects.

Inclusion criteria were: being regularly enrolled, aging 2 to 5 years, no gender restriction, having only one full primary dentition, and to having the informed consent form signed by parents/guardians. All 72 public municipal pre-schools in the city were invited to take part in the study, and only 11 accepted. The total number of children aged 2 to 5 years in each school was verified, and the sample was defined proportionally to the number of children enrolled (Figure 1). That is, the school with the largest number of children had the biggest samples. Children who had the informed consent form signed were randomly assigned to the study according to proportions previously established. Of three examiners, two were randomly selected for data collection at each institution.

Exclusion criteria were: children with permanent teeth erupted, who would not behave cooperatively for clinical examination, who were absent on the day of clinical appointment, or had cognitive and/or motor deficits reported by the preschool management.

Calibration had two steps: theoretical, carried out in two moments with a 15-day interval, which involved discussion of diagnosis criteria for dental trauma^24^ and esthetic impairment by analyzing photographs. Then clinical step, performed by three examiners who evaluated children aging 2 to 5 years in two occasions, with 7 to 14-day intervals between them. Kappa coefficient for inter- and intra-examiner agreement was >0.70 (Table 1).

**Chart 1:**
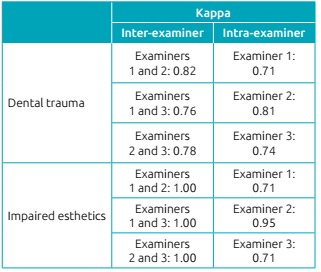
Results of Kappa coefficient for inter- and intra-examiner agreement regarding dental trauma and esthetic impairment.

The pilot study was conducted to test methodology and to understand the instruments. Twenty-seven preschool children from a municipal day care center participated. Those who participated in both the calibration and pilot study were not included in the main sample. The pilot allowed us to improve the clinical examination dynamics, making children’s acceptance easier.

Non-clinical data collection was made by application of two questionnaires to parents through children’s school journals in two steps. First, the questionnaire was aimed at the evaluation of OHRQoL; the Brazilian version of Early Childhood Oral Health Impact Scale (B-ECOHIS) was used.^16^ This questionnaire has two sections: child and family impact. Thirteen questions comprise six domains:

- Four domains in child-impact section: symptoms (one item), function (four items), psychological (two items), and self-image/social interaction (two items);
- Two domains in family-impact section: parental distress (two items) and family function (two items).

The category of answers was sorted in: 0=never, 1=hardly ever, 2=occasionally, 3=frequently, 4=very often, 5=do not know. Impact on quality of life was characterized by at least one of the questions being answered with“frequently”or“very often”.^19^ Questionnaires with more than two questions with answer “do not know” in the child-impact section (CIS) and one in the family-impact section (FIS) were excluded from the sample.^14^

In the second stage, after clinical exams, a questionnaire on socioeconomic data was sent to the families of children. Gender, birth date, head of household, and questions for economic classification of the Brazilian Association of Research Companies (ABEP) were covered.^20^ There were also questions related to the trauma event: “Has your child ever had a trauma on a milk tooth?” If the answer was positive, following questions were regarding the age of the child in the event, where and how it happened, and whether they were assisted by a dentist. All questions had answer options to choose from.

Data collection was made at the day care centers, also in two stages. The first step was to analyze esthetic commitment having the child seated facing the examiner at a conversation distance, without lip manipulation or artificial lighting, and being asked to smile. Esthetic impairment was accounted for when color change in the crown, fracture of more than half of the crown or dental absence (avulsion) were found in anterior superior teeth.^21^ ^,^ ^22^ For all other situations, esthetic impairment was considered absent.

In the second stage, clinical examination was performed to evaluate dental trauma, having the child seated facing the examiner, through direct visual observation of the oral cavity and the use of artificial lighting (LED light flashlight). Examination was made using gauze pads and a clinical mirror. The changes were noted in a specific clinical file. All biosafety standards were complied with (use of gloves, caps, and disposable masks).^23^

Evaluation of trauma was based on the criteria established by Andreasen and Andreasen^24^, which is based on the system adopted by the World Health Organization (WHO).^23^ Such classification includes enamel fracture, enamel and dentin fracture, and tooth absence (in cases of avulsion or early exodontia). In case of doubt regarding early exodontia due to sequelae of trauma and/or avulsion, clinical data were confronted with information passed on by parents/guardians or by children themselves. When identified upon clinical examination, lateral luxation and intrusion trauma were classified as “other traumas”. Presence of fistula/abscess and changes in crown color were also observed.^2^

The project was approved by the Ethics Committee of *Universidade Federal de Santa Catarina* (UFSC) (N. 343,658) and by the Municipal Secretary of Education of Florianopolis. Guardians of preschool children also had to sign the informed consent form.

Simple descriptive statistics were used to determine sample characteristics and to show the distribution of B-ECOHIS items. The socioeconomic variables were calculated according to ABEP criteria, using a scoring system.^20^ The Statistical Package for Social Sciences (SPSS for Windows, version 21.0, SPSS Inc., Chicago, IL, United States) was used for data processing. Chi-square and Fisher tests were applied to check associations between trauma and gender, age, quality of life and esthetic impairment. The level of significance was set at 5%.

## RESULTS

The total number of participants was 192. A descriptive analysis of variables is shown in Table 1. The response rates of the quality of life questionnaire and the second survey were, respectively, 100% and 51.5%. Educational level of the family head was ≥8 years in 83% of cases, and the family income was ≤3 minimum wages in 56.8% of households. In addition to socioeconomic data, parents/guardians answered the questionnaire about DT and only 14.7% of them knew about their children’s trauma events; 66.6% of these had occurred at home due to falls (47.6%).

**Table 1:**
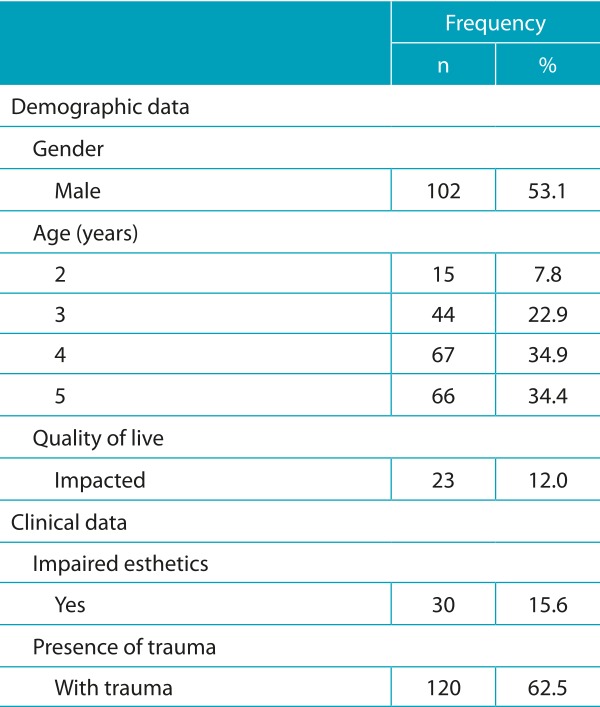
Descriptive analysis of variables (n=192).

Of 192 children, 120 (62.5%) had dental trauma, the most affected teeth being the right upper central incisor (35.7%), followed by the left upper central incisor (32.1%). In total, 2,304 upper and lower anterior teeth were evaluated, out of which 214 (9.2%) had some type of trauma. Among the evaluated teeth, 147 (68.7%) had enamel trauma; 37 (17.3% had both enamel and dentin trauma; 12 (5.6%), had crown color change; 6 (2.8%) were fistulae/abscesses; 3 (1.4%) dental absences; and 9 (4.2%) had other kinds of trauma. The maxillary central incisors were the most affected teeth (20.5%).

A descriptive analysis of B-ECOHIS showed that, after all parents of the participating children answered the questionnaire, the child-impact section had items related to pain (11.9%), difficulty eating (9.3%), difficulty sleeping (6.7%) and irritability (6.7%) as the most cited ones. In family-impact section, items associated with feelings (being bored/upset) (13.5%) and guilt (13.5%) were mentioned more frequently (as “sometimes”, “frequently”, and “very often”).

For best analysis of results, the variable trauma was dichotomized as: absent (n=143, no trauma and enamel trauma)^25^ and present (n=49; enamel and dentin trauma, tooth absence, fistula/abscess, lateral luxation, and intrusion). When DT was associated with gender, age and esthetic impairment, none of the variables had a significant association.

Data presented in Table 2 indicate no significant association between B-ECOHIS - child-impact section, family-impact section, and domains - and dental trauma, but association between quality of life and esthetic impairment was shown significant when tested. A relation between esthetic impairment and oral limitations was also pointed out.

**Table 2:**
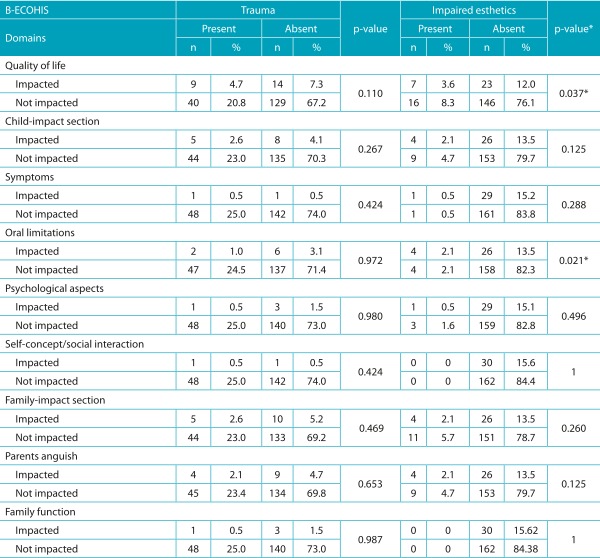
Relation of the Brazilian version of Early Childhood Oral Health Impact Scales - child-impact and family-impact sections, and domains - to trauma and impaired esthetics in preschool children.

B-ECOHIS: Brazilian version of *Early Childhood Oral Health Impact Scales*; *Chi-square test.

Table 3 shows non-significant association between quality of life and trauma (enamel trauma, enamel and dentin trauma, crown color change, fistula, tooth absence, other traumas). In 192 children examined, 120 had had a type of trauma. Thus, Table 4 shows the association between esthetic impairment and trauma. Association between esthetic impairment and crown color change was relevant. In 63.6% of children, color change was not seen at a normal talk distance; only when the lips were manipulated and the clinical mirror and light were used. These data emphasize the different perception of esthetics from a dentist’s and from the social interaction’s points of view.

**Table 3:**
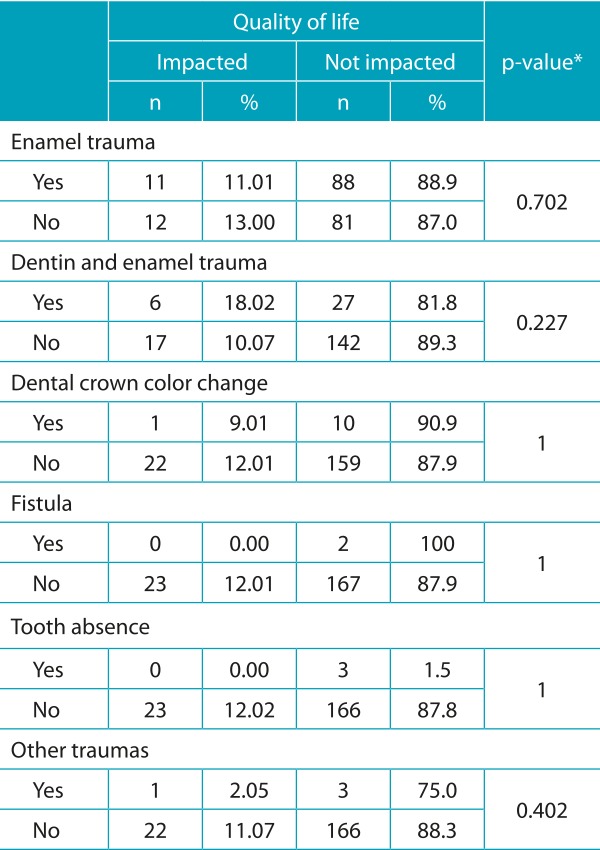
Association between quality of life and types of trauma.

*Chi-square test.

**Table 4:**
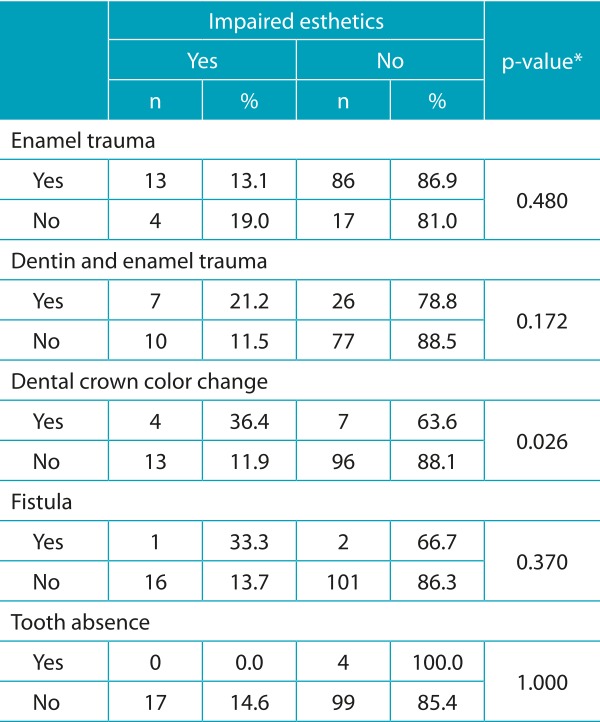
Association between trauma and impaired esthetics (n=120).

*Chi-square test.

## DISCUSSION

In this study, high occurrence of trauma in the children evaluated was shown. Other studies had the following rates of occurrence: 14,7%,^3^ 34,6%,^4^ 41,4%,^8^ 30%^26^ e 49,4%.^27^ Differences can be related to some studies being carried out in dental care centers and, therefore, the need for treatment of children studies may have influenced the results. Also, the criteria for evaluating dental trauma are different. In our study, for example, the classification by Andreasen and Andreasen^23^ ^,^ ^24^ was used with modifications such as crown color change^2^ ^,^ ^27^ and fistula/abscess. Although crown color change was not included in scale suggested by Andreasen and Andreasen,^24^ it is an important clinical variable when it comes to determining dental trauma history and it may also influence esthetics.

The variable trauma had the highest prevalence of enamel fracture, followed by enamel and dentin fracture, and crown color change. Such results are similar to previous findings,^27^ where enamel fracture was the most common outcome (50.6%), followed by crown color change (25.8%) and enamel and dentin fracture (14.4%). The study by Siqueira et al.^4^ also had enamel fracture as the most common outcome of trauma, with a 17% prevalence, followed by crown color change, with 11.2%. The most affected teeth in our study were the maxillary central incisors, with 88.4% of traumas, which coincides with the findings by Viegas et al.,^27^ who observed 62.8% of prevalence for these teeth. Soft-tissue lesions, dislocations or sub-luxations that might occur at the time of trauma cannot be seen, though. It limits clinical examination, as it is not able to assess the trauma in its full extent or its impact on quality of life.^28^

In the present investigation, items with the highest prevalence in the child-impact section of the B-ECOHIS were “related to pain” and “difficulty eating some types of food”, once dental trauma causes pain and sensitivity. The items with the highest incidence in the family-impact section were “feeling of guilt” and “feeling distressed/upset”, which corroborates other studies’ findings.^3^ ^,^ ^27^ ^,^ ^29^ Symptoms reported are compatible with dental trauma, and parents usually worry about their children, so they feel guilty about their oral problems, considering that pain is acknowledged by them when reported by children.

The presence of trauma did not influence negatively on children’s quality of life, corroborating some literature studies.^4^ ^,^ ^13^ ^,^ ^27^ Other studies^3^ ^,^ ^8^ ^,^ ^30^ showed a negative impact on quality of life caused by trauma. In the present study, answers “frequently” and “very often” were considered cutoff points to assess negative impact on quality of life. Siqueira et al.^4^ considered “occasionally”, “frequently” and “very often” as sign of quality of life impacted. Abano et al. and Viegas et al.^26^ ^,^ ^27^, in turn, used scores of questions as cutoff points. These differences in methodology make it difficult to compare the studies.

The esthetic impairment resulting from trauma was related to negative impact on quality of life and to oral limitations in the child-impact section. The literature lacks studies that associate trauma or esthetic impairment with quality of life in primary teeth, although pre-school children attribute behavioral characteristics to other children based on the attractiveness of their appearance.^31^ A study^32^ suggested that +3-year-old children are quite aware of the esthetic value of their dentition, including missing or darkened teeth, and this leads parents to seek dentists.

When esthetic impairment and crown color change were linked, the result was significant. Change in color goes unnoticed by the eyes of other individual, but when the focus of attention is directed to mouth of the child affected, it becomes the main reason of negative perceptions.^33^ It is known that dentoalveolar trauma is very common in childhood and often results in esthetic impairment.^8^ Surprisingly, no epidemiological investigation has ever been made on the social impact caused by changes in anterior primary teeth, and little is known about how children feel when they have incisors with esthetic changes or about the possible social, psychological, and emotional consequences on their behavior.^10^

It is worth mentioning, however, that the present study presented two important limitations, one of them being small number of schools participating, which compromises the external validity of the research. Therefore, a broader study with a larger number of schools is suggested to expand the application of results. The second limitation was the low rate of response in the second survey sent over to parents/guardians. Although it is expected in studies involving questionnaires, sending two questionnaires in different moments impaired data collection.

Despite these limitations, we can conclude that esthetic impairment by dental trauma had a negative impact on the quality of life of preschool children and their relatives. Predictors of negative impact on quality of life may vary among populations and should be taken into account in processes of decision-making processes for allocation of resources to health programs.^4^ Further studies with larger sample sizes are recommended in order to reach beyond this population; longitudinal designs are also suggested, to establish a causal relationship between the associations identified.
